# Repurposing flufenamic acid as a putative PmrB-directed adjuvant to restore colistin activity in *Klebsiella pneumoniae*

**DOI:** 10.1128/spectrum.03620-25

**Published:** 2026-03-17

**Authors:** Chuleeporn Phanus-umporn, Nuttapat Anuwongcharoen, Chawalit Chatupheeraphat, Noramon Kaewsai, Warawan Eiamphungporn

**Affiliations:** 1 Department of Community Medical Technology, Faculty of Medical Technology, Mahidol University, Nakhon Pathom, Thailand; 2 Center for Research Innovation and Biomedical Informatics, Faculty of Medical Technology, Mahidol University, Nakhon Pathom, Thailand; 3 Department of Clinical Microbiology and Applied Technology, Faculty of Medical Technology, Mahidol University, Bangkok, Thailand; JMI Laboratories, North Liberty, Iowa, USA

**Keywords:** PmrB, colistin resistance, *Klebsiella pneumoniae*, virtual screening, antibiotic adjuvant, flufenamic acid

## Abstract

Antimicrobial resistance in gram-negative bacteria has renewed reliance on colistin, yet resistance to this last-line agent is increasing. In *Klebsiella pneumoniae*, activation of the PmrA/PmrB two-component system remodels lipid A and reduces colistin binding, motivating the sensor kinase PmrB as an adjuvant target. Here, we combined computational drug repurposing with experimental validation to identify colistin potentiators. High-throughput virtual screening of DrugBank using integrated ligand- and structure-based approaches shortlisted five approved drugs (mebendazole, flurbiprofen, tirbanibulin, flufenamic acid, and netarsudil) for experimental evaluation. *In vitro* assays against colistin-resistant *K. pneumoniae* showed flufenamic acid most robustly resensitized resistant isolates and suppressed the emergence of additional resistance under combination exposure. Mechanistic studies indicated that the colistin-flufenamic acid combination increased membrane permeability and significantly downregulated PmrA/PmrB-regulated genes (*pmrC* and *arnT*). Molecular docking and molecular dynamics simulations further supported a plausible interaction of flufenamic acid with the PmrB ATPase region, and representative PmrB substitutions (D150Y, T157P, and R256G) did not alter the predicted binding mode. Together, these data suggest that modulation of the PmrA/PmrB pathway may contribute to colistin potentiation and demonstrate a target-guided repurposing-to-validation framework for antibiotic adjuvant discovery in colistin-resistant *K. pneumoniae*.

**IMPORTANCE:**

Colistin is one of the last remaining treatment options for multidrug-resistant *Klebsiella pneumoniae* infections, but resistance to this drug is rising worldwide. In this study, we used a PrmB-informed, computer-guided drug repurposing workflow followed by *in vitro* validation to identify approved compounds that can potentiate colistin activity against colistin-resistant *K. pneumoniae*. This approach independently prioritized flufenamic acid as the most robust colistin enhancer among the screened candidates, restoring colistin activity and reducing the emergence of additional resistance under combination exposure. Mechanistically, our findings are consistent with modulation of the PmrB/PmrA resistance pathway, supporting bacterial histidine-kinase signaling as a promising direction for antibiotic adjuvant development. Overall, this study highlights a practical and scalable framework for discovering resistance-targeted adjuvants to help protect last-resort antibiotics and improve treatment options for difficult-to-treat infections.

## INTRODUCTION

Antimicrobial resistance is a major threat to global health, driving substantial economic losses and a growing public-health crisis (1, 2). The challenge is particularly acute for infections caused by multidrug-resistant gram-negative bacteria (GNB) (3). With resistance spreading and few new agents available, colistin has been reintroduced as a last-resort therapy (4). Unsurprisingly, this renewed use has been followed by increasing reports of colistin-resistant (COL-R) *Klebsiella pneumoniae*, *Escherichia coli*, *Pseudomonas aeruginosa*, and *Acinetobacter baumannii* worldwide (5), underscoring the need for strategies that can restore the activity of existing drugs. Compared with *de novo* antibiotic discovery, combination therapy offers a faster, safer, and often more economical path to improved outcomes (6). One promising approach is the use of antibiotic adjuvants, which are co-administered compounds that enhance antibiotic efficacy by improving uptake, counteracting resistance pathways, or optimizing pharmacokinetics (7). In the case of colistin, pairing with an appropriate adjuvant could resensitize resistant bacteria.

Among GNB, *K. pneumoniae* is a leading cause of severe opportunistic infections and exhibits alarming, rising resistance (8). Colistin resistance in *K. pneumoniae* arises primarily through two main mechanisms (9). The most common is chromosomal mutations in key regulatory genes like the PmrA/PmrB and PhoP/PhoQ two-component systems and their negative regulator, MgrB (9, 10). These mutations remodel the bacterial outer membrane, specifically the lipopolysaccharide (LPS) layer, which prevents colistin from binding to and disrupting the cell. The second mechanism is the acquisition of mobile colistin resistance (*mcr*) genes carried on plasmids. These genes can be easily transferred between bacteria, allowing resistance to spread rapidly (11). Both mechanisms ultimately work by altering the LPS to achieve resistance to colistin, making these infections incredibly difficult to treat.

Within this framework, PmrB, a membrane histidine kinase, is a compelling candidate adjuvant target because it controls a major chromosomal pathway of colistin non-susceptibility in Enterobacterales, including *K. pneumoniae* (12). As a sensor kinase of the PmrA/PmrB two-component system, PmrB autophosphorylates and transfers phosphate to the response regulator PmrA, which upregulates lipid A modification genes; these changes reduce the net negative charge of LPS and thereby diminish colistin binding and bactericidal activity (13). Because histidine kinases initiate these resistance-associated signaling cascades, they represent attractive targets for adjuvant development. Indeed, small molecules reported to interact with the ATP-binding region of PmrB (or related histidine kinases) can attenuate PmrAB-mediated resistance phenotypes in *A. baumannii*, providing a conceptual blueprint for PmrB focused-inhibitor discovery (14). Accordingly, compounds that interfere with PmrB kinase function may resensitize bacteria to colistin in strains where PmrAB signaling contributes to the resistant phenotype.

This rationale motivates the use of computational approaches to prioritize potential colistin adjuvants directed toward PmrB-associated resistance pathways. Such strategies address major limitations of conventional drug discovery, including high costs, lengthy development timelines, and low success rates. Virtual screening enables rapid interrogation of large chemical libraries and prioritization of candidates for experimental validation. Ligand-based approaches, such as chemical similarity searching, identify novel compounds by referencing known bioactive molecules, whereas structure-based methods, particularly molecular docking, simulate candidate interactions within target binding sites and estimate binding poses and affinities (15, 16). Taken together, these complementary approaches can efficiently enrich for candidates with plausible target engagement.

In this study, we performed high-throughput virtual screening of DrugBank using a PmrB-focused strategy and shortlisted five approved drugs (mebendazole, flurbiprofen, tirbanibulin, flufenamic acid, and netarsudil) by combining chemical similarity analysis and molecular docking. These candidates were then evaluated *in vitro* against colistin-resistant *K. pneumoniae*. Under identical testing conditions, flufenamic acid was the most robust colistin potentiator, resensitizing resistant isolates and suppressing the emergence of additional resistance under combination exposure. Mechanistic studies in *K. pneumoniae* using N-phenyl-1-naphthylamine (NPN) and propidium iodide (PI) assays and RT-qPCR indicated that the combination enhances membrane damage and downregulates PmrA/PmrB-regulated lipid A modification genes (*pmrC* and *arnT*). Consistent with a target-engagement hypothesis, molecular docking and molecular dynamics (MD) simulations supported a plausible, stable interaction of flufenamic acid with the PmrB ATPase region, and representative PmrB substitutions (D150Y, T157P, and R256G) did not significantly impact the predicted binding mode. Therefore, these findings are consistent with modulation of the PmrA/PmrB pathway contributing to colistin potentiation in *K. pneumoniae* and illustrate a scalable target-guided repurposing-to-validation framework for antibiotic adjuvant discovery.

## MATERIALS AND METHODS

### Data collection and preparation

A data set of drugs was obtained from the DrugBank database (version 5.1.9), an online resource providing comprehensive molecular, pharmacological, and pharmaceutical information on drugs and their targets (17). The DrugBank database was curated through multiple filtration steps to generate the final set of compounds. Initially, we retained only those entries explicitly classified as approved or veterinary approved, thereby excluding compounds with a status of withdrawn, illicit, or nutraceutical, which are unsuitable for clinical use. Subsequently, the compounds were filtered according to Lipinski’s Rule of Five, a key predictive model for oral bioavailability. This step ensured that all retained compounds possessed critical drug-like characteristics, specifically molecular weight <500 Da, the octanol-water partition coefficient (LogP) ≤5, number of hydrogen bond (H-bond) donors ≤5, and number of hydrogen bond acceptors ≤10 (18). Finally, compounds lacking complete or well-defined chemical structures were excluded, a critical step to ensure the generation of reliable molecular fingerprints and the accuracy of ligand-based virtual screening.

### Similarity search for ligand-based virtual screening

The preprocessed DrugBank data set was utilized to perform chemical similarity analysis with ATP, which serves as the natural ligand of PmrB (19). Molecular fingerprints were generated using the FP3, as implemented in the Open Babel toolkit (20). FP3 encodes the presence of predefined structural fragments (substructures), making it suitable for capturing key functional groups and molecular features. The similarity between ATP and each drug was quantified using the Tanimoto coefficient, a widely used metric for comparing binary molecular fingerprints. As illustrated in Formula 1, the Tanimoto coefficient measures the overlap between fingerprint vectors, where higher values indicate greater structural similarity:


(1) $$\text{ Tanimoto coefficient }(AB)=\frac{AB}{\;A+B-AB}$$

where *AB* is the set of common bits in the fingerprints of molecules *A* and *B*. *AB* is the set of common bits of fingerprints of both molecule A and B. Thus, the Tanimoto coefficient is the ratio of the cardinality of the intersection and the cardinality of the union of two fingerprints. Tanimoto score ranges from 0 (when the fingerprints have no bits in common) to 1 (when the fingerprints are identical) (21). A threshold of 0.5 was rigorously applied as the minimum cutoff for retaining potential drug candidates for further analysis.

### Receptor and ligand library preparation

The full-length amino acid sequence of *K. pneumoniae* PmrB (NCBI reference: WP_004886129.1) served as the query for a Position-Specific Iterated BLAST search against the Protein Data Bank (PDB). This search aimed to identify a suitable structural template for homology modeling. The crystal structure of a PmrB homolog (PDB ID: 4BIW) was selected due to its high sequence identity and, critically, the presence of a co-crystallized ATP analog, confirming a functionally relevant active site conformation. To enhance model accuracy and reduce computational demands associated with modeling the full-length protein, the model was focused on the catalytic and DHp domains (residues 130–365). The homology model was constructed using MODELLER (22), which generates a three-dimensional structure based on the alignment with the template. The refined PmrB homology model was prepared as the receptor for docking studies using AutoDockTools by adding polar hydrogen atoms and assigning Gasteiger partial charges, which are essential steps for accurately calculating electrostatic interactions. In parallel, a set of ligands obtained from ligand-based virtual screening was prepared in the same manner as the receptor to ensure consistency.

### Structure-based virtual screening

Structure-based virtual screening was carried out using AutoDock Vina (23). The docking search space was explicitly defined by centering a grid box over the predicted ATP-binding (ATPase) region of the PmrB structural model. The grid box was configured with the following parameters: a center of (*x*, *y*, *z*) = [63.943, −51.864, 3.489] and dimensions of (*x*, *y*, *z*) = [18.75, 22.5, 22.5] Å. To ensure a more rigorous exploration of ligand conformations, the exhaustiveness parameter was increased to 16, while all other docking parameters were maintained at their default settings. Each compound in the final library was docked into the prepared PmrB receptor. The resulting poses were evaluated using AutoDock Vina’s scoring function, which calculates a binding energy (kcal/mol) as an estimate of binding affinity. In addition, the ligand efficiency (LE) matrix was calculated to normalize the predicted binding affinity of the candidates by their size (24). This allows us to compare the results with the ATP, which is larger in size and has a greater van der Waals volume. The LE can be calculated by Formula 2:


(2) $$LE=\frac{-\Delta G}{\;N}$$

where ∆*G* is the docking score in kilocalories per mole, and *N* is the number of heavy atoms. After ranking all compounds based on these scores, the top five with the lowest (most favorable) scores were selected as candidates for subsequent experimental validation.

### Bacterial isolates and reagents

Using anonymized clinical isolates, the study involved no interaction with human subjects and no access to identifiable private information. A total of six non-duplicate COL-R *K. pneumoniae* isolates were obtained from the bacterial repository of the Department of Clinical Microbiology and Applied Technology, Faculty of Medical Technology, Mahidol University. Isolates were identified as *K. pneumoniae* by conventional biochemical tests as previously described (25), including Gram stain morphology and the following profile: oxidase negative, non-motile, indole negative, methyl red negative, Voges-Proskauer positive, citrate positive, malonate positive, urease positive, phenylalanine deaminase negative, glucose fermentation with gas production, lysine decarboxylase positive, and ornithine decarboxylase negative. *K. pneumoniae* ATCC 700603 served as a quality control strain in this study. Flufenamic acid and colistin sulfate were obtained from Sigma-Aldrich (St. Louis, MO, USA) and Chem-Impex International (Wood Dale, IL, USA), respectively. Other candidate drugs were purchased from MedChemExpress (Monmouth Junction, NJ, USA). CAMHB (cation-adjusted Mueller-Hinton II broth) was from Becton Dickinson (Franklin Lakes, NJ, USA), and additional chemicals were purchased from Sigma-Aldrich. Colistin solutions were prepared using sterile Milli-Q water before the experiments. Flufenamic acid stock solutions were dissolved in dimethyl sulfoxide.

### Checkerboard assays

Drug interactions were assessed by checkerboard microdilution (26). CAMHB with graded concentrations was dispensed in 96-well U-bottom plates: the candidate drug across rows A–H (0–256 µg/mL) and colistin across columns 1–11 (0–1,024 µg/mL). Column 1 and row H provided single-agent MICs; A1 was the growth control. Log-phase cultures were adjusted to 0.5 McFarland, diluted 1:100 to 10^6^ CFU/mL, and inoculated into wells. After incubation at 37°C for 20–24 h, MICs were read visually as the lowest concentrations preventing growth. Synergy of the combination was quantified by the fractional inhibitory concentration index (FICI). The FICI was calculated using Formula 3. Interactions were interpreted as follows: synergy for FICI ≤0.5; no interaction for 0.5 < FICI ≥ 4; antagonism for FICI >4 (27).


 (3) $$FICI=\frac{\text{ MIC of drug A in the combination }}{\text{ MIC of drug A alone }}+\frac{\text{ MIC of drug B in the combination }}{\text{ MIC of drug B alone }}$$

For off-scale MICs (MIC >highest concentration tested), the MIC was set to the highest tested concentration (64 µg/mL) for FICI calculation; thus, reported FICI values represent conservative upper bounds (true FICI <reported).

### Time-kill assays

Synergy was further examined by time-kill studies on two COL-R isolates as described previously, with minor modifications (28). Flufenamic acid and colistin were tested alone and in combination at concentrations guided by checkerboard results. Bacteria were exposed to flufenamic acid and colistin alone or in combination at 10^5^ CFU/mL. The cultures were shaken at 180 rpm at 37°C, and bacterial counts were performed on Mueller-Hinton agar at 0, 1, 2, 4, 6, 8, and 24 h. Killing curves were created by plotting the viable bacterial counts over time. A synergistic effect was defined as a reduction of more than 2 log10 in CFU per milliliter after 24 h of combination treatment, compared to the CFU per milliliter reduction observed with either colistin or flufenamic acid alone.

### Emergence of resistance/serial passage assays

The influence of flufenamic acid on the development of acquired resistance to colistin was assessed using continuously passaged cultures as described previously (29). Briefly, the MICs of either colistin or a combination of colistin and flufenamic acid against colistin-susceptible (COL-S) and colistin-resistant *K. pneumoniae* strains were determined on the first day. After incubating for 24 h, the bacterial cells growing at the highest concentration (1/2× MIC) were harvested as the working inoculum for the subsequent experiment day. An additional MIC assay was then conducted using this inoculum, and the MIC was determined the next day. This was repeated daily for 30 days. MICs were recorded each day and plotted as MIC (µg/mL) relative to day 1.

### Membrane permeability assays

The NPN uptake assay was performed to evaluate the outer membrane permeability as described elsewhere (30). In brief, COL-R *K. pneumoniae* isolates were grown to mid-log phase in CAMHB medium. Cultures were then treated with 1/4× MIC colistin, 16 µg/mL flufenamic acid, and the combination for 2 h. Untreated cells served as the negative control. The cells were collected by centrifugation, washed twice with assay buffer (5 mM HEPES, 5 mM glucose, pH 7.4), and resuspended in the same buffer to achieve a final OD_600_ of 0.2. NPN was dissolved in acetone at a concentration of 50 mM and then diluted in HEPES buffer to prepare a 50 µM working stock. Subsequently, NPN and the cell suspension were mixed in a 96-well optical-bottom black plate (SPL Life Sciences, Pocheon-si, South Korea), resulting in a final NPN concentration of 10 µM. Fluorescence was measured using a microplate reader (Ensight, Multimode; Perkin Elmer, Waltham, MA, USA) with excitation and emission wavelengths set to 350 and 420 nm, respectively. A control sample consisting of cell suspension without NPN was used to subtract background fluorescence.

The PI staining assay was used to investigate the inner membrane integrity as previously described (31). Cultures of COL-R strains in the mid-log phase were treated with 1/4× MIC colistin, 16 µg/mL flufenamic acid, and the combination for 2 h. Cells heat-treated at 80°C for 2 h and untreated cells were used as positive and negative controls, respectively. The cells were harvested by centrifugation, washed twice with assay buffer (10 mM PBS, pH 7.4), and resuspended in the buffer to an OD_600_ of 0.2. The PI fluorescent dye was dissolved in PBS buffer at a concentration of 50 µg/mL. The PI solution was then added to the cell suspension in a 96-well optical-bottom black plate, resulting in a final PI concentration of 10 µg/mL. Fluorescence intensity was measured using a microplate reader with an excitation wavelength of 488 nm and an emission wavelength of 630 nm.

### RT-qPCR analysis

RT-qPCR was used to measure the expression of the *pmrB*, *pmrC*, and *arnT* (also referred to as *pmrK*) genes, using the primers listed in Table 1. Bacterial cultures were grown to mid-log phase in CAMHB and further incubated with colistin (1/4× MIC) or flufenamic acid alone (16 μg/mL) or in combination with both drugs for 4 h. Cells without any drug treatment were used as controls. Cell pellets were collected by centrifugation, and total RNA was isolated using the RNAprep Pure Cell/Bacteria Kit (Tiangen, Beijing, China) according to the manufacturer’s protocol. Then, RNA samples were treated with DNase enzyme to remove contaminating DNA using TURBO DNA-free Kit (Thermo Fisher Scientific, Waltham, MA, USA). The treated RNA was converted to cDNA using iScript cDNA Synthesis kit (Bio-Rad, Hercules, CA, USA). The extracted RNA was adjusted to the same concentration during the DNA elimination process before reverse transcription. Subsequently, RT-qPCR was carried out using Luna Universal qPCR Master Mix (New England Biolabs, Ipswich, MA, USA). The amplification was performed in 20 μL reaction mixtures containing 10 μL master mix, 10 μM forward and reverse primers, and 2 μL template. The *rpoD* gene encoding a housekeeping sigma factor was used as a reference gene. The reacting condition was set as a two-step method as follows: pre-denaturation at 95°C for 30 s, 40 cycles consisting of denaturation at 95°C for 5 s, and annealing at 60°C for 34 s. The *rpoD* gene was used to standardize the expression levels, and the data of mRNA relative expression were analyzed by the 2^−△△Ct^ method, as previously described (32).

**TABLE 1:** RT-qPCR primers used in this study

Gene	Primer	Sequence (5′–3′)	Amplicon size (bp)	Reference
*pmrB*	pmrB-RT-F	GGC CGT CGT CTC TGG CGA TG	78	(33)
	pmrB-RT-R	GGG CTG TAG CGG TGA GCA TTC		
*pmrC*	pmrC-RT-F	CTC TCG CCT CGT TCC TGA A	79	(34)
	pmrC-RT-R	CGG AGT GGT GTC GAG GAT A		
*arnT*	pmrK-RT-F	CGC TGA ATA TGC TCG ACC CAG AAG	108	(33)
	pmrK-RT-R	GCT GGC GGT AAT CGT CTG TAC G		
*rpoD*	rpoD-RT-F	TCC GGT GCA TAT GAT TGA GA	105	(35)
	rpoD-RT-R	ATA CGC TCA GCC AGC TCT TC		

### Molecular dynamics simulation of PmrB and flufenamic acid complex

Following experimental prioritization of flufenamic acid, additional computational studies were performed to evaluate a plausible binding mode and characterize its predicted interaction with PmrB. As the initial model was a truncated form, a new full-length model of *K. pneumoniae* PmrB was constructed using a ligand-guided homology modeling approach previously described (36). Briefly, an ensemble of 1,000 full-length PmrB models was generated. Each model was then subjected to molecular docking with flufenamic acid using AutoDockFR (37), employing the same parameters as the reference study. The PmrB model that yielded the lowest docking score with flufenamic acid was selected as the most representative conformation for further analysis.

To investigate the stability and dynamics of the predicted interaction, all-atom MD simulations were performed on the selected PmrB-flufenamic acid complex. The simulation protocol mirrored the methodology outlined in the previous work (36), utilizing GROMACS (38) with the CHARMM36 force field. The complex was solvated in a cubic box with TIP3P water molecules, maintaining a minimum distance of 10 Å from the box edges. Counterions were added to neutralize the system’s overall charge. The system then underwent a two-stage equilibration process, starting with a 100 ps NVT (canonical ensemble) equilibration at 300 K, followed by a 100 ps NPT (isothermal-isobaric ensemble) equilibration at 300 K and 1 bar. Subsequently, 200 ns MD simulations were conducted in duplicate to analyze the system’s dynamic behavior. Following the simulations, binding free energies were estimated using the molecular mechanics generalized Born surface area (MM/GBSA) method as implemented in the gmx_MMPBSA tool (39). To identify key residues driving the interaction, a per-residue energy decomposition analysis was also performed.

### *In silico* mutagenesis

To investigate the effect of mutation presence in the experimental strains, the PmrB-ATP and flufenamic acid complexes were mutated using FoldX (40) to generate the mutant structures of D150Y, T157P, and R256G. The molecular docking, using AutodockFR, was carried out to evaluate the change in docking score of ATP and flufenamic acid against mutants compared to the wild type.

### Statistical analysis

All experiments were conducted with a minimum of three independent assays. The quantitative results are expressed as the mean ± standard deviation. Statistical analysis was performed using GraphPad Prism 8 (GraphPad Software, San Diego, CA, USA), with data analyzed by Student’s *t*-test and one-way analysis of variance. A *P* value of <0.05 was considered statistically significant.

## RESULTS

### Ligand-based virtual screening prioritizes FDA-approved drugs for potential PmrB-targeted colistin potentiation

A set of 11,912 compounds was initially retrieved from the DrugBank database. After filtering to retain only approved and veterinary-approved drugs and to exclude withdrawn, illicit, nutraceutical, or structurally incomplete entries, 2,466 compounds were obtained. Subsequently, the application of Lipinski’s Rule of Five reduced the data set to 1,768 compounds. The final curated data were used to identify compounds exhibiting high structural similarity to ATP, with potential relevance to PmrB-associated adjuvant activity.

To evaluate chemical similarity, compounds were encoded using the FP3 fingerprint implemented in the Open Babel toolkit. Similarity to ATP was assessed using the Tanimoto coefficient, with a cutoff of 0.5. A total of 671 compounds exceeding this threshold were identified. These selected compounds were compiled into a ligand data set and subsequently subjected to molecular docking to evaluate their predicted binding to the PmrB ATPase region (based on the PmrB structural model used in this study).

### Structure-based virtual screening prioritizes FDA-approved drugs predicted to interact with the PmrB ATP-binding site

To prioritize FDA-approved compounds with predicted interaction potential toward *K. pneumoniae* PmrB, structure-based virtual screening was performed by molecular docking against the ATP-binding site of the previously generated PmrB homology model using AutoDock Vina (23). The screening results were ranked based on the predicted binding affinity, which estimates the docking score in kilocalories per mole. From this analysis, the top five compounds emerged as the most promising candidates. These were identified as mebendazole (DrugBank ID: DB00643, Vina score: −8.854 kcal/mol), flurbiprofen (DB00712, −8.646 kcal/mol), tirbanibulin (DB06137, −8.524 kcal/mol), flufenamic acid (DB02266, −8.478 kcal/mol), and netarsudil (DB13931, −8.358 kcal/mol). Although the docking score of these compounds is inferior to that of ATP, it can be noted that flurbiprofen, flufenamic acid, and mebendazole exhibited a more favorable LE value of −0.480, −0.424, and −0.402, respectively, as compared to ATP (Table 2). These top-ranked hits, representing a diverse set of chemical scaffolds, were selected for experimental validation. A summary of docking score and ligand efficiencies for the top five candidates, along with their chemical structures, is shown in Table 2.

**TABLE 2:** Summary of top five FDA-approved drugs ranked by docking score at the ATPase region of the *K. pneumoniae* PmrB model

Rank	DrugBank ID	Name (Tanimoto coefficient)	Docking score (kcal/mol)	Ligand efficiency (kcal/mol/heavy atom)
1	DB00643	Mebendazole (0.50)	−8.854	−0.402
2	DB00712	Flurbiprofen (0.50)	−8.646	−0.480
3	DB06137	Tirbanibulin (0.50)	−8.524	−0.266
4	DB02266	Flufenamic acid (0.58)	−8.478	−0.424
5	DB13931	Netarsudil (0.50)	−8.358	−0.246
Reference	DB00171	Adenosine triphosphate (1.00)	−8.936	−0.288

### Synergy of colistin with candidate drugs by checkerboard testing

The checkerboard assay was used to determine the antimicrobial interaction between colistin and five candidate drugs. Because all candidate compounds showed off-scale MICs when tested alone (MIC >64 µg/mL), FICI values were calculated using 64 µg/mL as a conservative upper-bound estimate. Under this approach, only the colistin-flufenamic acid combination met the synergy criterion across all isolates (FICI ≤0.5) and produced substantial reductions in colistin MICs, shifting them into the susceptible range according to our interpretive criteria and restoring an *in vitro* colistin-susceptible phenotype under the conditions tested (Table 3). No other drugs met the synergy criterion. Notably, the FICI-based interaction outcomes for all candidates and the previously characterized mechanisms of colistin resistance for each isolate are summarized in Table 4, including mutations in *mgrB*, *pmrA*, and *pmrB*, with no detection of *mcr-1* (41).

**TABLE 3:** The MIC and FICI values of flufenamic acid (FFA) combined with colistin (COL) against COL-R *K. pneumoniae*

Isolate number	MIC of monotherapy (µg/mL)		MIC of combination (µg/mL)		FICI	Interpretation
	COL	FFA^*a*^	COL	FFA		
KP-03	32	>64	2	4	0.125	Synergy
KP-04	16	>64	2	4	0.188	Synergy
KP-07	64	>64	2	4	0.094	Synergy
KP-08	128	>64	2	8	0.141	Synergy
KP-13	256	>64	1	4	0.066	Synergy
KP-15	32	>64	1	4	0.094	Synergy

^*a*^ MICs reported as >64 µg/mL were set to 64 µg/mL for FICI calculations (conservative upper bound, true FICI <reported).

**TABLE 4:** Checkerboard synergy of candidate drugs with colistin and associated resistance mechanisms

Isolate	Mechanism of colistin resistance	FICI between colistin and candidates^*a*^				
		Mebendazole	Flurbiprofen	Tirbanibulin	Flufenamic acid	Netarsudil
KP-03	PmrA (E57G), MgrB (truncated protein, 29 aa)	2.000	2.000	2.000	0.125	1.016
KP-04	PmrB (R256G)	2.000	2.000	2.000	0.188	2.000
KP-07	PmrA (E57G)	2.000	1.500	2.000	0.094	1.016
KP-08	PmrA (E57G) and *ΔpmrA452-461*, PmrB (D150Y)	2.000	2.000	0.516	0.141	1.016
KP-13	PmrA (E57G) and *ΔpmrA452-461*, PmrB (D150Y)	2.000	1.250	2.000	0.066	1.125
KP-15	PmrA (E57G), PmrB (T157P)	2.000	0.750	2.000	0.094	1.000

^*a*^ The interaction was assessed using the FICI: synergy, FICI ≤0.5; no interaction, 0.5 < FICI ≤ 4; antagonism, FICI >4.

### Synergistic activity testing by time-kill assays

**Fig 1:**
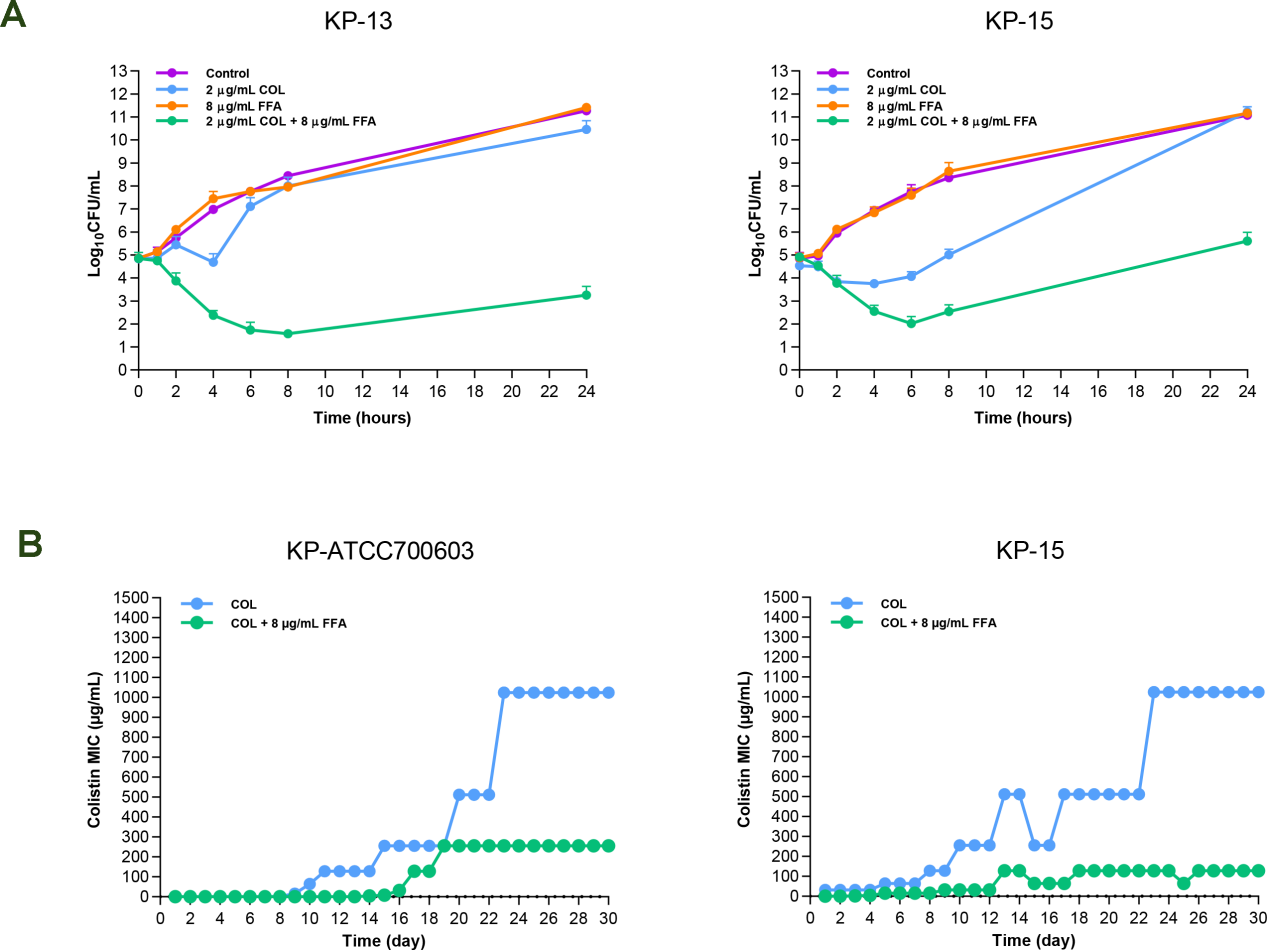
Flufenamic acid enhances colistin activity against colistin-resistant (COL-R) *K. pneumoniae* and attenuates resistance emergence *in vitro*. (**A**) Time-kill kinetics for COL-R isolates treated with colistin (COL), flufenamic acid (FFA) alone, or the combination; the combination yields ≥2 log10 CFU/mL reductions at 24 h relative to either monotherapy. (**B**) Resistance development assay showing that co-exposure to flufenamic acid suppresses the *in vitro* increase colistin MICs in both COL-S and COL-R strains.

To corroborate the synergy between flufenamic acid and colistin, time-kill assays were performed on two representative Col-R *K. pneumoniae* isolates. Drug concentrations were selected from checkerboard conditions that met the synergy criterion (upper-bound FICI <0.5). As shown in Fig. 1A, flufenamic acid or colistin alone produced killing curves comparable to untreated controls. In contrast, the colistin-flufenamic acid combination achieved more rapid bactericidal activity, yielding ≥2 log10 CFU/mL reductions in viable counts at 24 h compared with the most active single agent. Thus, the bactericidal effect of colistin was substantially enhanced in combination with flufenamic acid.

### Flufenamic acid restricts the evolution of colistin resistance

To evaluate whether flufenamic acid suppresses the emergence of colistin resistance, resistance development assays were performed using *K. pneumoniae* strains that were COL-S (ATCC 700603) or COL-R (KP-15). In the COL-S strain, the initial colistin MIC was 0.5 µg/mL under both conditions, indicating that flufenamic acid did not potentiate colistin activity in this colistin-susceptible strain. In contrast, in the early passages of the COL-R strain, the MIC measured under the combination condition was lower than colistin alone (1 µg/mL vs 32 µg/mL), confirming that flufenamic acid potentiated colistin activity in this resistant background. During evolution in both strains, the increase in colistin MIC under the combination condition was delayed compared with colistin alone. By passage 30, the MIC increase was markedly greater in the colistin alone than in the combination, indicating that flufenamic acid delayed the onset and reduced the magnitude of resistance development (Fig. 1B).

### Flufenamic acid enhances the membrane-damaging ability of colistin

**Fig 2:**
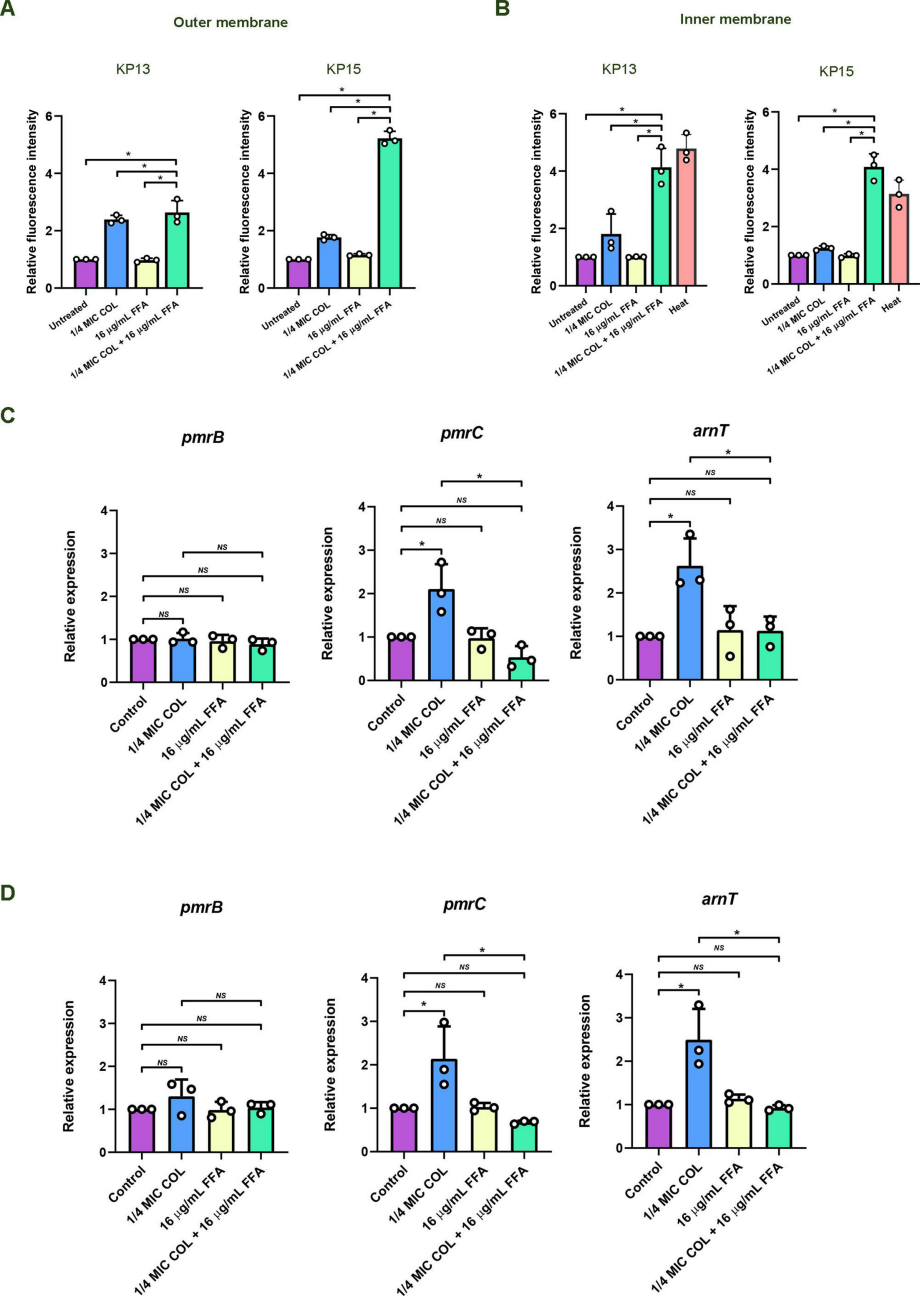
Effects of colistin-flufenamic acid (FFA) combination on membrane permeability and expression of PmrAB-regulated lipid A modification genes. (**A and B**) Outer and inner membrane permeability in COL-R *K. pneumoniae* isolates KP-13 and KP-15, assessed by NPN (**A**) and PI (**B**) fluorescence after exposure to 16 µg/mL FFA, a subinhibitory concentration of colistin (COL), or the combination. (**C and D**) Relative expression of *pmrB*, *pmrC*, and *arnT* in KP-13 (**C**) and KP-15 (**D**) under the same conditions. Data were analyzed by one-way analysis of variance. *P value <0.05; *NS*, not statistically significant.

To visually assess the combined therapy’s effect on cell membrane permeability, outer membrane and inner membrane permeability were measured using NPN and PI, respectively. Results showed that colistin at subinhibitory concentrations led to a slight increase in NPN uptake in KP-13 and KP-15 COL-R strains (Fig. 2A), but flufenamic acid alone had no significant effect. Crucially, the combination of colistin and flufenamic acid resulted in a significant increase in NPN uptake, clearly demonstrating enhanced outer membrane permeability. Regarding inner membrane permeability, colistin alone had a minimal impact on PI uptake, and flufenamic acid alone showed no altered fluorescence intensity (Fig. 2B). However, the combined treatment dramatically increased fluorescence intensity, strongly suggesting inner membrane disruption. These findings collectively establish that flufenamic acid potentiates colistin activity by significantly enhancing both outer membrane and inner membrane damage.

### Combination of colistin with flufenamic acid downregulates the *pmrC* and *arnT* expression

Virtual screening and molecular docking suggested a plausible interaction between flufenamic acid and PmrB, raising the hypothesis that flufenamic acid could modulate the PmrAB two-component system and its regulon. To investigate pathway-level effects, we performed RT-qPCR analysis of *pmrB*, *pmrC*, and *arnT* gene expression in KP-13 and KP-15 isolates exposed to colistin, flufenamic acid, or their combination. In KP-13, subinhibitory colistin alone increased *pmrC* and *arnT* expression by 2.10- and 2.63-fold, respectively, compared to the untreated control (Fig. 2C), consistent with induction of lipid A modification genes associated with colistin resistance. Flufenamic acid alone did not significantly change the expression of either gene. In contrast, the combination of colistin and flufenamic acid significantly decreased *pmrC* and *arnT* expression by 3.94- and 2.33-fold, respectively, compared to the colistin, indicating downregulation of these resistance genes. A similar pattern was observed in KP-15 (Fig. 2D). Colistin alone increased *pmrC* and *arnT* expression by 2.14- and 2.49-fold, while the combination decreased them by 3.16- and 2.70-fold, respectively, compared to the colistin. The flufenamic acid alone again showed no significant change. Notably, *pmrB* expression remained relatively unchanged across all treatments in both isolates. Overall, these transcriptional data indicate that flufenamic acid counteracts colistin-induced upregulation of the PmrAB-regulated lipid A modification genes *pmrC* and *arnT* under combination exposure. Together with the *in silico* results, the findings are consistent with modulation of the PmrA/PmrB resistance pathway, although direct target engagement and inhibition of PmrB were not experimentally demonstrated in this study.

### Predicted binding mode of the PmrB-flufenamic acid complex

**Fig 3:**
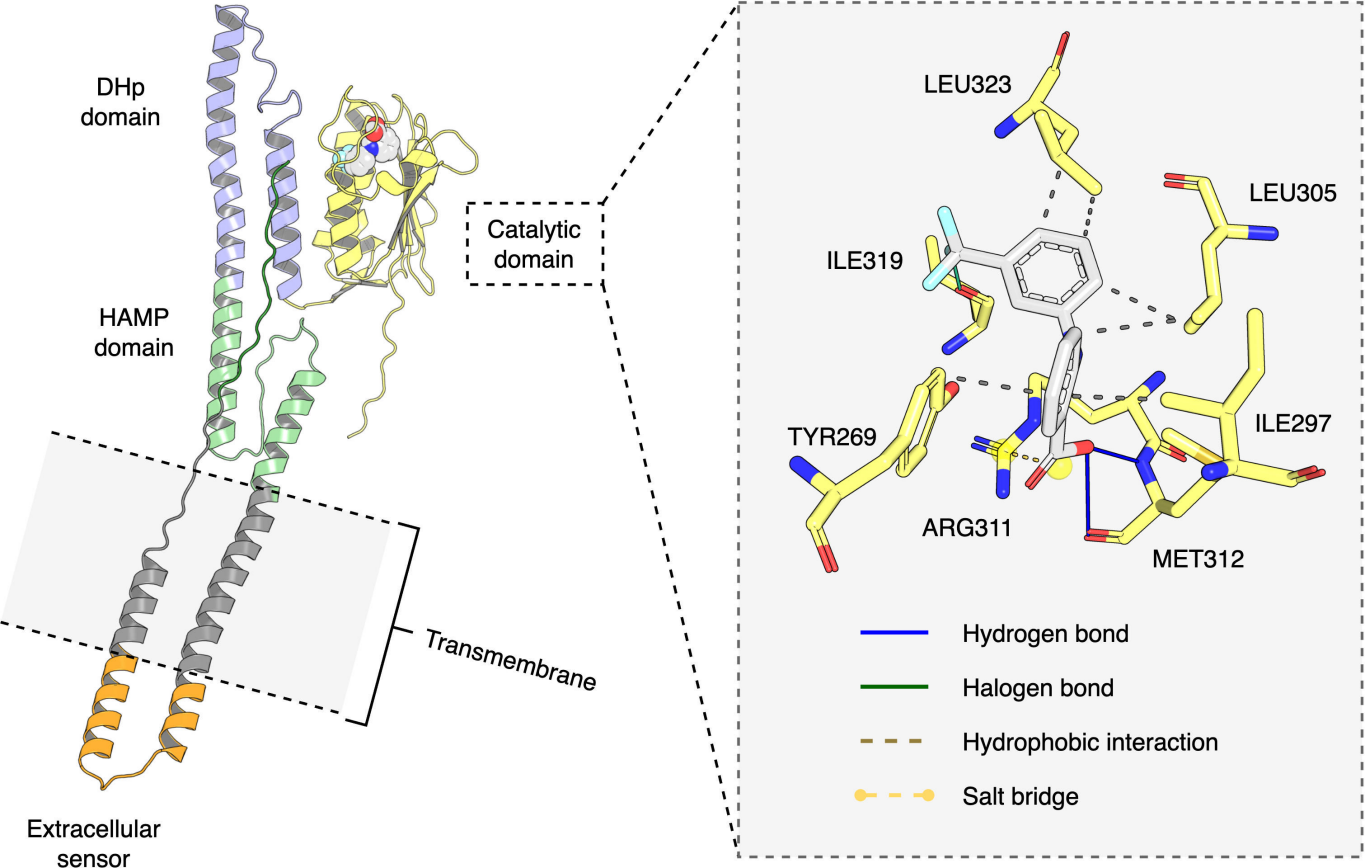
Predicted protein-ligand interactions between *K. pneumoniae* PmrB and flufenamic acid from molecular docking. The full-length modeled PmrB-flufenamic acid complex is shown in the left panel, and the right panel represents the predicted interacting residues within the ATPase region.

Based on its synergistic activity with colistin, flufenamic acid was selected for additional computational analysis to define a plausible binding pose within the PmrB kinase domain. A new, full-length *K. pneumoniae* PmrB model was generated using a ligand-guided approach because the initial truncated model used for screening lacked complete structural context for subsequent dynamic simulations. From an ensemble of full-length models, the conformation yielding the most favorable docking score with flufenamic acid was selected as a representative structure for interaction analysis. Inspection of the top-ranked docking pose using the Protein-Ligand Interaction Profiler identified a network of predicted interactions positioning flufenamic acid within the ATP-binding site, as illustrated in Fig. 3. The pose is anchored by a salt bridge between the carboxylate group of the benzoic acid moiety of flufenamic acid and the side chain of ARG311, a key residue within the catalytic domain, at a distance of 3.38 Å. This electrostatic interaction is supported by hydrogen bonding contact involving the MET312 residue from the ATP-lid loop at the catalytic domains. The carboxylate oxygen atoms act as both donors and acceptors for hydrogen bonds from the side chain of the MET312 main chain (2.56–2.90 Å). Furthermore, the trifluoromethyl group of flufenamic acid forms a halogen bond with the guanidinium side chain of ARG311 (3.38 Å), which may further constrain ligand orientation. The complex is also stabilized by numerous non-polar interactions. Extensive hydrophobic contacts were identified with residues spanning both the ATPase lid loop and catalytic domains, including TYR296, ILE297, LEU305, and LEU323. Altogether, these predicted electrostatic, hydrogen-bonding interactions, together with halogen-bonding and hydrophobic contacts, provide a plausible structural rationale for how flufenamic acid could associate with the PmrB ATPase region in the modeled complex.

### Effects of PmrB mutations

To investigate whether PmrB substitutions present in our clinical isolates could influence the predicted interaction, mutant structures were generated from the wild-type PmrB model using FoldX. The predicted binding of flufenamic acid and ATP to wild-type and mutant PmrB proteins was then assessed by AutoDockFR, and the resulting docking scores are shown in Table 5. For wild-type PmrB, ATP, the natural substrate, yielded a docking score of −9.6 ± 1.3 kcal/mol, whereas flufenamic acid yielded a docking score of −8.1 ± 0.1 kcal/mol. Across the three substitutions examined, docking predicted reduced ATP binding relative to wild type. The D150Y mutation had the most significant impact, with a predicted ATP docking score of −8.1 ± 0.2 kcal/mol, which is 1.5 kcal/mol weaker than the wild type. The T157P and R256G substitutions yielded an ATP docking score of −9.1 kcal/mol, corresponding to a 0.4 kcal/mol decrease compared with wild type. In contrast, these substitutions had minimal predicted impact on flufenamic acid docking. The D150Y variant yielded −7.9 ± 0.1 kcal/mol, approximately 0.1 kcal/mol less favorable than wild type, while the T157P and R256G variants yielded −8.0 ± 0.1 kcal/mol, with differences of +0.1 kcal/mol and +0.0 kcal/mol, respectively.

**TABLE 5:** Predicted effects of PmrB substitutions on docking score of flufenamic acid and adenosine triphosphate (ATP) (PmrB substrate) using AutoDockFR

Ligand	Mutation	Docking score (kcal/mol)	Score difference (kcal/mol)
Flufenamic acid	D150Y	−7.9 ± 0.1	+0.1
	T157P	−8.0 ± 0.0	+0.1
	R256G	−8.0 ± 0.1	+0.0
	Wild type	−8.1 ± 0.1	–^*a*^
ATP	D150Y	−8.1 ± 0.2	+1.5
	T157P	−9.1 ± 0.1	+0.4
	R256G	−9.1 ± 1.2	+0.4
	Wild type	−9.6 ± 1.3	–

^*a*^ –, not applicable.

### Molecular dynamics simulations and binding free energy analysis

**Fig 4:**
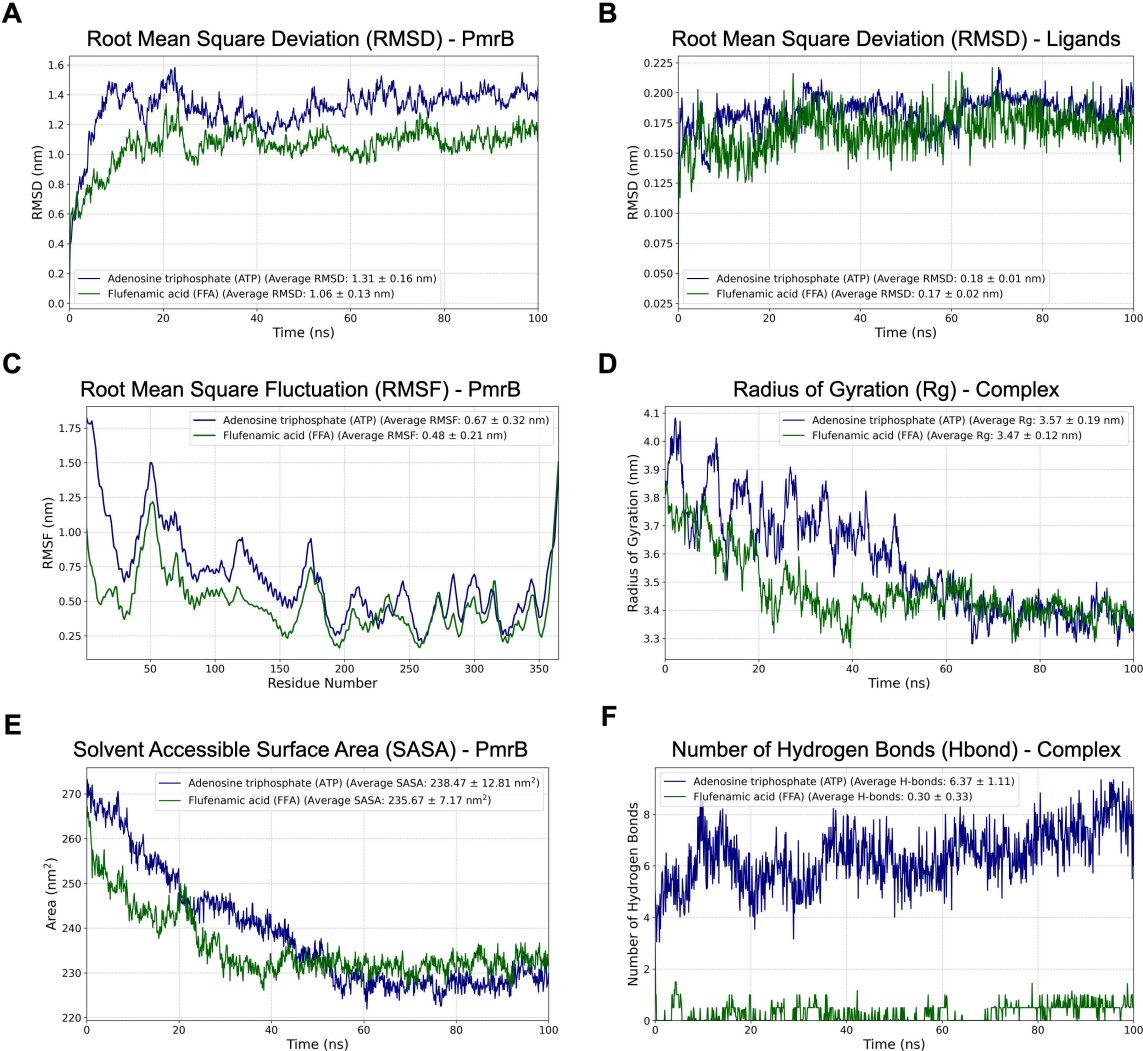
Structural behavior and stability of modeled PmrB complexes from duplicate molecular dynamics simulations. (**A**) Root mean square deviation (RMSD) of PmrB protein backbones over the simulations. (**B**) RMSD of ATP and flufenamic acid (FFA) within the PmrB ATPase region. (**C**) Root mean square fluctuation (RMSF) values across the PmrB backbone’s residues, indicating flexible regions over simulation. (**D**) Radius of gyration (Rg), showing protein compactness over time. (**E**) Solvent accessible surface area (SASA) of the PmrB, reflecting the exposed surface area. (**F**) Number of hydrogen bonds between PmrB and ATP or flufenamic acid during the simulations.

To assess the stability of the predicted binding pose and the dynamics of the modeled PmrB-flufenamic acid complex, duplicate 100 ns all-atom MD simulations were conducted. Global MD trajectory analyses were consistent with stable complexes over the simulation timescale (Fig. 4). Protein structural stability was evaluated by calculating the root mean square deviation (RMSD) of backbone atoms relative to the initial structure. The protein reached an apparent equilibrium after 60 ns, with backbone RMSD values averaging 1.31 and 1.06 Å for the ATP and flufenamic acid, respectively, suggesting that the modeled protein maintained overall fold without significant conformational changes during the simulations. The stability of the ligand pose was assessed by ligand RMSD that fluctuated minimally around 0.18 and 0.17 Å for ATP and flufenamic acid, respectively, indicating that the ligands remained in a similar orientation within the modeled binding pocket. Further analysis of the root mean square fluctuation (RMSF) per residue indicated that the catalytic domain (residues 206–365), especially the core residues defining the ATP-binding site, remained relatively static, while the remaining structures had higher mobility and flexibility. The radius of gyration (Rg) of the protein remained consistent after equilibrium with the average Rg for ATP and flufenamic acid of 3.57 and 3.47, respectively, confirming that the complex maintained its overall compactness and stability. Similar patterns were observed for solvent accessible surface area (SASA), with comparable average values for ATP- and flufenamic acid-bound simulations (238.47 and 235.67, respectively), suggesting no major change in overall solvent exposure. The number of H-bonds exhibited a divergent pattern between ATP and flufenamic acid binding. The ATP exhibited significantly higher H-bond of 6.37, while the flufenamic acid exhibited dramatically lower H-bond of 0.30. Although the docking score obtained from the molecular docking was ranked in close proximity, these results suggested that the ATP and flufenamic acid bind via different driving forces, in which ATP mainly relies on the polar contact and electrostatic interaction, while the flufenamic acid depends on the non-polar, π-cationic, and halogen-bond interaction. Overall, these MD-derived descriptors support the modeled stability of the PmrB-ligand complexes over the simulation timescale.

**Fig 5:**
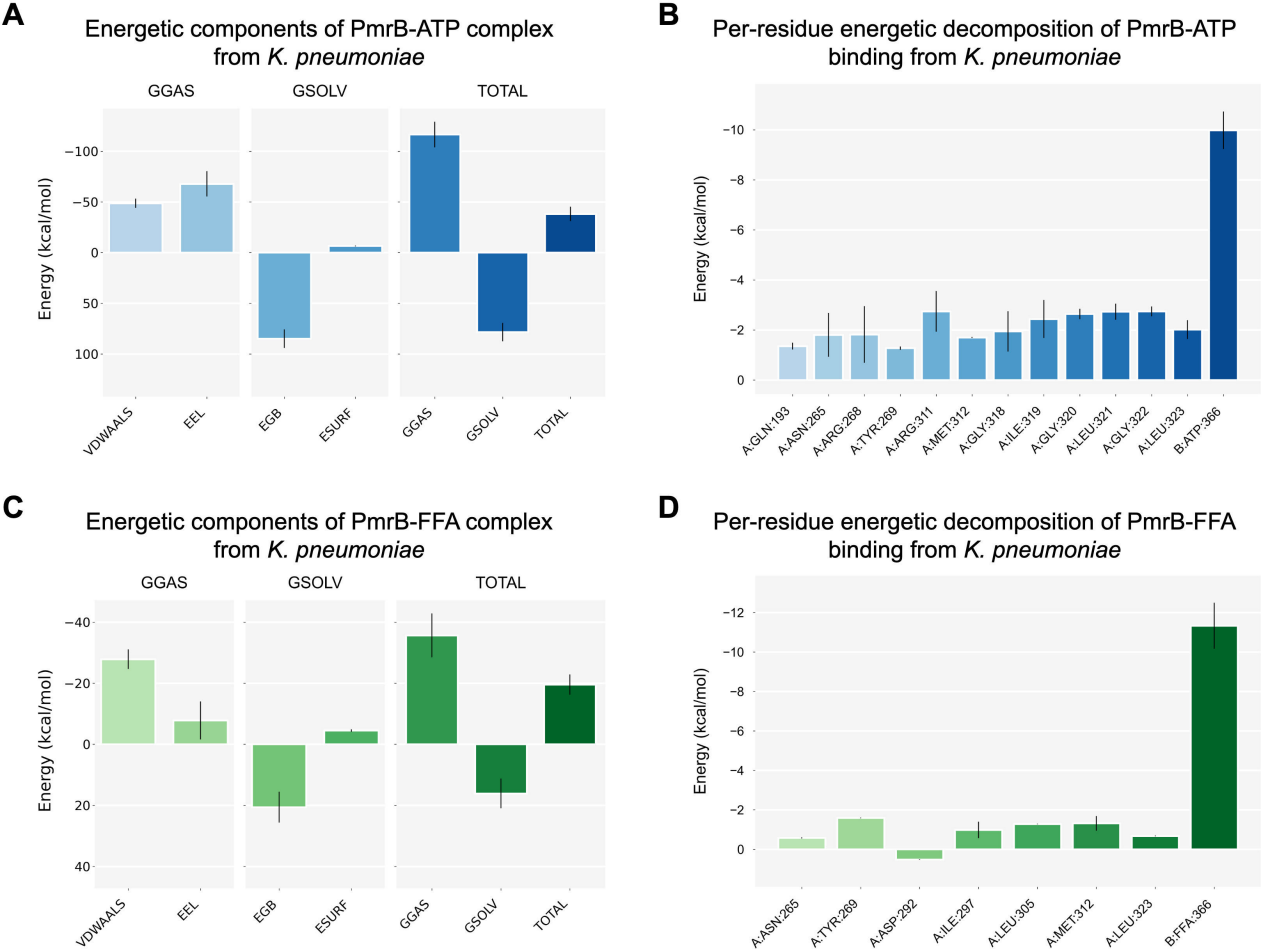
Estimated binding free energy components and per-residue energy decomposition from MM/GBSA calculations for modeled PmrB-ATP and PmrB-flufenamic acid (FFA) complexes. Left panels (**A and C**) show MM/GBSA energy components for PmrB-ATP (**A**) and the PmrB-FFA (**C**), including VDWAALS, EEL, polar solvation (EGB), and non-polar solvation (ESURF) terms. Right panels (**B and D**) present per-residue decomposition analyses for ATP and FFA interactions.

To further estimate the strength of binding, the MM/GBSA method was used to calculate the binding free energy (Δ*G*) from the simulation trajectories, as shown in Fig. 5 and Table 6. The ATP yielded a highly favorable Δ*G* of −38.14 ± 7.53 kcal/mol (Fig. 5A). The modeled PmrB-flufenamic acid complex yielded an estimated Δ*G* of −19.56 ± 3.46 kcal/mol (Fig. 5C), consistent with a favorable interaction in the ATPase-site model. Decomposition analysis of energetic components reveals different driving forces for these two ligands. For ATP, the interaction was characterized by a highly favorable gas-phase molecular mechanics energy (GGAS) of −116.47 kcal/mol, composed of strong van der Waals interaction energy (VDWAALS) and dominant electrostatic interaction energy (EEL) contributions of −48.65 kcal/mol and −67.82 kcal/mol, respectively. This favorable interaction is counteracted by a substantial unfavorable solvation free energy (GSOLV) of 78.33 kcal/mol. In contrast, flufenamic acid showed more modest results, with a GGAS energy of −35.65 kcal/mol. This interaction was driven primarily by VDWAALS forces of −27.84 kcal/mol and a much smaller EEL contribution of −7.81 kcal/mol, with a correspondingly lower unfavorable GSOLV energy of 16.08 kcal/mol.

**TABLE 6:** MM/GBSA-estimated binding free energy (∆*G*) for PmrB complexes with adenosine triphosphate (ATP) and flufenamic acid^*a*^

Energy terms	ATP			Flufenamic acid		
	∆*G* (kcal/mol)	SD (kcal/mol)	SEM (kcal/mol)	∆*G* (kcal/mol)	SD (kcal/mol)	SEM (kcal/mol)
VDWAALS	−48.65	4.57	0.03	−27.84	3.21	0.02
EEL	−67.82	19.92	0.14	−7.81	6.64	0.05
EGB	84.88	15.58	0.11	20.58	5.22	0.04
ESURF	−6.55	0.43	0.00	−4.50	0.40	0.00
GGAS	−116.47	19.52	0.14	−35.65	7.58	0.05
GSOLV	78.33	15.37	0.11	16.08	5.05	0.04
Total	−38.14	7.53	0.05	−19.56	3.46	0.02

^*a*^ Abbreviations: ∆*G*, average of binding free energy; EEL, electrostatic interaction energy; EGB, polar solvation energy using the Generalized Born model; ESURF, non-polar solvation energy; GGAS, gas-phase molecular mechanics energy; GSOLV, solvation free energy; SD, standard deviation; SEM, standard error of mean; VDWAALS, van der Waals interaction energy.

Further analysis using per-residue decomposition identified residues predicted to contribute most to each interaction. For ATP, several residues were found to be major contributors, with the most significant favorable contributions (less than −2.0 kcal/mol) from ARG311, ILE319, GLY320, LEU321, and GLY322, as depicted in Fig. 5B. For flufenamic acid, the individual residue contributions were weaker overall (greater than −2.0 kcal/mol), with the most stabilizing predicted contributions provided by TYR269, ILE297, LEU305, MET312, and LEU323, while ASP292 exhibited an unfavorable contribution, as shown in Fig. 5D. This residue is adjacent to the TYR269, and in the modeled complex, the negative charge of the ASP292 side chain may influence the local environment near TYR269, potentially reducing the favorability of flufenamic acid association at this site. Collectively, these simulations provide a computational description of a plausible interaction mode of flufenamic acid at the PmrB ATPase region, while recognizing that experimental target validation is required to confirm direct binding or inhibition.

## DISCUSSION

The global proliferation of antimicrobial resistance, particularly among MDR gram-negative pathogens, is recognized as a grave public-health threat (1, 3). Colistin has consequently been adopted as a last-resort agent for these infections; however, the rising prevalence of colistin resistance has prompted intensified efforts to identify compounds that can potentiate its activity or restore susceptibility. Since experimental high-throughput screening is time-consuming and costly, virtual screening provides a faster, cost-effective approach alternative to prioritize candidates for experimental validation (15, 42). Virtual screening has been applied to prioritize compounds predicted to interact with enzymes involved in lipid A modification and colistin resistance, including MCR-1 and ArnT (43, 44). A complementary strategy is to target upstream regulatory pathways, such as the PmrA/PmrB two-component system that contributes to activation of lipid A remodeling programs. Genetic disruption of *pmrB*, which encodes the sensor histidine kinase PmrB, has been reported to restore colistin efficacy and to increase susceptibility to additional antimicrobials (45). Accordingly, we performed ligand- and structure-based computational screening of the DrugBank library against the catalytic domain of a PmrB structural model to prioritize candidate colistin potentiators for experimental evaluation.

For computational approaches, the initial filtering strategy that retained only approved and veterinary-approved drugs offered significant advantages for repurposing as colistin adjuvants. By prioritizing compounds with established regulatory approval, safety profiles, and pharmacokinetic data, the risk of late-stage attrition can be minimized. This approach substantially shortens development timelines and reduces costs by comparison with *de novo* drug discovery (46, 47). Lipinski’s Rule of Five filtering ensured that the remaining molecules possessed physicochemical properties favorable for oral bioavailability, including appropriate molecular weight, lipophilicity, and hydrogen-bonding profiles. Together, these dual filtering steps enhanced the likelihood of identifying clinically relevant compounds for subsequent evaluations (18). After that, a chemical similarity search was performed as a ligand-based approach to prioritize potential colistin adjuvants using ATP as the reference molecule. This method relies on the assumption that molecules sharing key chemical features may exhibit related bioactivity profiles (48). The Tanimoto coefficient, based on the Open Babel FP3 fingerprint, is widely used for structural similarity assessment and quantifies shared fingerprint features between two molecules (49, 50). The result identified that 671 compounds exceeded the 0.5 similarity threshold. A cutoff of 0.5 was selected as a criteria filter, as it is widely used in large-scale cheminformatics studies and represents the level at which structural overlap becomes statistically meaningful, effectively removing irrelevant molecules while retaining structurally analogous compounds (51). Furthermore, molecular docking was performed to complement the chemical similarity screen by predicting ligand poses within the ATP-binding region of the PmrB structural model and ranking candidates by docking scores. While docking scores do not constitute experimental evidence of binding or inhibition, they are useful for prioritizing compounds with plausible target engagement for follow-up testing (52). Using this integrated ligand- and structure-based strategy, mebendazole, flurbiprofen, tirbanibulin, flufenamic acid, and netarsudil ranked among the top candidates by docking scores and ligand efficiencies. These candidates share aromatic ring features that partially resemble ATP substructures and may facilitate accommodation within the modeled ATPase pocket. Therefore, from an initial library of more than 10,000 compounds, integrated computational filtering and docking refined the selection to five candidates for experimental validation.

Synergistic activity with colistin was observed only for flufenamic acid among the five shortlisted candidates, as supported by our *in vitro* experiments. This observation is especially remarkable, as flufenamic acid exhibited a Tanimoto similarity score of 0.58 compared with ATP, slightly higher than the 0.50 observed for the other candidates and ranked fourth in molecular docking with a docking score of −8.478 kcal/mol, a value not significantly different from the other compounds. These findings emphasize that computational approaches primarily serve as prioritization tools that reduce large chemical libraries into tractable subsets. Consequently, only a small number of compounds are typically expected to demonstrate the desired activity during experimental validation. In this regard, the identification of a single compound from the five top-ranked computational candidates that translated into a confirmed biological hit should be considered a meaningful success for the virtual screening strategy, consistent with previous reports showing that experimental validation often yields only a limited fraction of active compounds from large-scale computational screening (53–55).

The computational results suggest that PmrB associates with its native substrate, ATP, with approximately twice the estimated binding free energy magnitude compared with flufenamic acid (based on MM/GBSA). The analysis of the energetic components and per-residue contributions provides a detailed rationale for this difference in computed binding strength. The binding of ATP is predominantly enthalpically driven, mainly through EEL interactions (−67.82 kcal/mol). This finding is supported by per-residue decomposition, which identifies ARG311 as a key contributor during complex formation. Analysis of key-interacting residues and their moieties of preference suggests that the positively charged guanidinium group of this arginine residue forms a strong salt bridge with the negatively charged phosphate groups of ATP. This electrostatic attraction, combined with substantial van der Waals contacts, is sufficient to overcome the massive energetic penalty (GSOLV = 78.33 kcal/mol) associated with desolvating the highly charged ATP and the polar residues in the binding site. On the other hand, the predicted binding of flufenamic acid was primarily driven by hydrophobic and van der Waals interactions, whereas the EEL contribution to its binding is minimal (−7.81 kcal/mol). The VDWAALS forces (−27.84 kcal/mol) account for the majority of its GGAS contribution. This is correlated with the chemical nature of flufenamic acid, which is composed of two largely hydrophobic aromatic moieties. Per-residue analysis revealed key contributions from hydrophobic (ILE297, LEU305, and MET312) and aromatic (TYR269) residues, likely forming hydrophobic contacts and potential π-stacking interactions. The desolvation penalty for flufenamic acid is much smaller (GSOLV = 16.08 kcal/mol), reflecting its less polar nature. Interestingly, the trifluoromethyl group presented in flufenamic acid may promote halogen-bond-like contacts with ILE319 during the simulation, suggesting a possible anchoring interaction within the ATPase binding site. Nevertheless, this residue was not highlighted in the per-residue decomposition, likely because traditional molecular mechanics force fields used in gmx_MMPBSA do not capture halogen-bonding by default (56). Interestingly, MET312 and LEU323 contribute favorably to the binding of both ligands. This indicates the importance of these residues within the binding pocket, similar to the previous study (36), by providing a hydrogen bond donor to the adenine and phosphate group of ATP, respectively, and by serving as a hydrophobic surface that stabilizes the aromatic rings of flufenamic acid. These findings further support that the PmrB ATPase site can accommodate these two ligands via distinct physical mechanisms in which ATP binding was driven by electrostatic forces, whereas flufenamic acid binding is dominated by non-polar, shape-complementarity interactions.

Flufenamic acid is a non-steroidal anti-inflammatory drug (NSAID) used to manage pain and inflammation, particularly in rheumatic conditions (57). Its primary pharmacological effect is achieved through inhibition of cyclooxygenase enzymes, which reduces prostaglandin synthesis. Beyond these established actions, flufenamic acid has shown *in vitro* antimicrobial activity against diverse pathogens, including methicillin-resistant *Staphylococcus aureus* (MRSA), *Neisseria gonorrhoeae*, and *Candida albicans* (58–60). *In silico* analyses have further supported antibacterial potential by demonstrating appreciable binding affinity of flufenamic acid to DNA gyrase in *Salmonella enterica* serovar Typhimurium, suggesting a plausible mechanism for antimicrobial action (61). Importantly, flufenamic acid has been reported to function as a colistin adjuvant, resensitizing colistin-resistant gram-negative bacteria such as *Escherichia coli*, *K. pneumoniae*, *A. baumannii*, and *P. aeruginosa* (62). In contrast to prior work emphasizing broad phenotypic activity, we apply a PmrB-informed selection strategy and provide complementary pathway-level mechanistic evidence in clinical COL-R *K. pneumoniae*, including dual NPN/PI permeability profiling and repression of PmrA/PmrB-regulated lipid A modification genes. Consistent with these observations, synergy was observed in our checkerboard assays across all COL-R *K. pneumoniae* isolates, with colistin MICs reduced to ≤2 µg/mL, a threshold consistent with widely used the CLSI/EUCAST breakpoint (63, 64). Time-kill assays corroborated these findings by showing enhanced bactericidal activity of colistin in the presence of flufenamic acid. Moreover, as additional support, a published study reported *in vivo* activity of the colistin-flufenamic acid combination, including improved survival in a *Galleria mellonella* infection model and reduced bacterial burdens in a murine infection model (62). Collectively, these data support flufenamic acid as a promising colistin-sensitizing adjuvant.

Notably, a previous study reported that an adjuvant can enhance colistin activity by increasing bacterial membrane permeability and that flufenamic acid alone increased inner membrane permeability in a dose-dependent manner in *P. aeruginosa* (62). However, our investigation of the colistin-flufenamic acid combination in COL-R *K. pneumoniae* yielded nuanced results. Using NPN and PI staining to assess outer and inner membrane permeability, respectively, we found that significant membrane disruption was caused by combined treatment with colistin and flufenamic acid, as evidenced by a notable increase in fluorescence intensity. This suggests that flufenamic acid potentiates colistin activity by enhancing damage to both bacterial membranes. Intriguingly, unlike the previous report, flufenamic acid alone at a fixed concentration of 16 µg/mL did not affect the membrane permeability of *K. pneumoniae* as measured by the PI signal. This discrepancy is likely due to the different experimental designs, as the previous study observed membrane permeability increases only at much higher flufenamic acid concentrations. Our findings are consistent with a mechanism in *K. pneumoniae* in which flufenamic acid primarily enhances colistin-mediated membrane damage rather than causing direct membrane disruption on its own at the concentrations tested.

Molecular docking simulations and transcriptional profiling were used to investigate how flufenamic acid restores colistin susceptibility. Docking simulations predicted that flufenamic acid can be accommodated within the catalytic pocket of PmrB and may engage key residues in the modeled ATPase site, consistent with a potential effect on PmrB function. Consistent with attenuation of the PmrAB regulon at the transcriptional level, exposure of COL-R *K. pneumoniae* to flufenamic acid (in combination with colistin) was associated with reduced expression of *pmrC* and *arnT*, which encode the lipid A phosphoethanolamine and aminoarabinose transferases, respectively, that diminish colistin binding (65). Prior studies have shown that certain colistin combinations can diminish PmrAB activity and downregulate its regulon (66, 67). In line with those reports, the most consistent transcriptional change observed here was repression of downstream lipid A modification genes rather than induction of *pmrB* itself, which is often modest and strain dependent (68–70). Taken together, these findings support a model in which flufenamic acid modulates the PmrAB pathway, thereby suppressing the lipid A modification program and facilitating preservation of colistin binding and activity.

Docking of ATP and flufenamic acid against wild-type and mutant PmrB models suggested that the three substitutions presented in our *in vitro* isolates (D150Y, T157P, and R256G) were associated with less favorable docking score for ATP, with D150Y showing the largest shift. In contrast, these substitutions had a negligible effect (±0.1 kcal/mol) on the docking score for flufenamic acid, suggesting that the modeled flufenamic acid pose is relatively tolerant to these substitutions and is dominated by non-polar contacts, consistent with molecular dynamics analysis. Therefore, these computational results provide a plausible explanation for our *in vitro* observation that flufenamic acid-mediated colistin potentiation was retained in isolates carrying these substitutions. Nevertheless, docking and molecular dynamics simulations provide only *in silico* support for a plausible interaction, and RT-qPCR repression of PmrA/PmrB-regulated genes (e.g., *pmrC* and *arnT*) reflects downstream pathway modulation rather than direct evidence of PmrB binding or autokinase inhibition. Definitive confirmation will require orthogonal validation, such as (i) biochemical kinase assays with purified or reconstituted PmrB (or its cytosolic kinase domain) to test inhibition of autophosphorylation and/or phosphotransfer to PmrA; (ii) direct binding measurements (thermal-shift analysis, surface plasmon resonance, or isothermal titration calorimetry); and/or (iii) genetic tests demonstrating loss or marked reduction of potentiation in a *pmrB* deletion background or in isogenic allelic-replacement strains carrying kinase-inactive PmrB variants. These experiments, particularly those requiring recombinant expression and functional reconstitution of a membrane histidine kinase, were beyond the scope of the present study. Accordingly, we interpret PmrB as a putative target and present PmrAB pathway engagement as a working mechanistic model that motivates future validation.

The capacity of flufenamic acid to limit the emergence of colistin resistance was evaluated using a resistance development assay in both COL-S and COL-R *K. pneumoniae* strains. Cultures were passaged daily for 30 days under colistin pressure with or without flufenamic acid, and MICs were determined at each interval. Co-exposure to flufenamic acid markedly attenuated the rise in colistin MICs in both strain backgrounds relative to colistin alone. These findings indicate that combining flufenamic acid with colistin may not only enhance treatment of ongoing infections but also reduce the propensity for resistance amplification or acquisition during prolonged exposure under the conditions tested. To the best of our knowledge, this is among the first reports in *K. pneumoniae* showing that this specific drug combination can suppress resistance development *in vitro*, consistent with prior reports that certain colistin adjuvants reduce resistance emergence (41, 71, 72).

From a pharmacokinetic perspective, the potential to use a lower dose of a last-resort antibiotic is a major clinical benefit. A reduced colistin dosage could minimize its serious adverse effects, such as nephrotoxicity and neurotoxicity. In principle, lowering antibiotic exposure could also reduce selective pressure that drives resistance development. This strategy is promising because the *in vitro* concentrations associated with potentiation appear to fall within a range that may be achievable *in vivo*. In our study, flufenamic acid potentiated colistin at 4–16 µg/mL, which overlaps with reported peak plasma concentration (typically 6–20 µg/mL) (73). While this overlap is encouraging, translating *in vitro* synergy to clinical benefit will depend on factors such as protein binding, free-drug exposure at the infection site, and tissue penetration. It is also noteworthy that flufenamic acid is reported to preferentially accumulate in human skin tissues, relevant to its topical NSAID use, whereas systematic data on distribution to other tissues remain limited (74). Accordingly, further pharmacokinetic/pharmacodynamic studies will be important to define exposure targets and dosing strategies for combination therapy.

Safety considerations also support continued evaluation of flufenamic acid as a potential colistin adjuvant. In prior *in vitro* work, no appreciable cytotoxicity to mammalian cells was observed at a flufenamic acid concentration of 62.5 μg/mL, which corresponded to the MIC against MRSA (60) and exceeded the concentrations used here for colistin potentiation against colistin-resistant *K. pneumoniae. In vivo*, efficacy and an acceptable biosafety profile for the colistin-flufenamic acid combination were demonstrated in a murine infection model (62). Although a precise lethal dose for flufenamic acid has not been established, toxicologic data from overdose cases involving the related fenamate mefenamic acid indicate that serious adverse effects such as seizures typically occur after single ingestions of approximately 2.5 g or more, producing serum levels far above the concentrations associated with *in vitro* potentiation (75). The separation between effective potentiating concentrations and doses linked to toxicity suggests a favorable therapeutic window. Taken together, these findings indicate that flufenamic acid, at exposures aligned with reported pharmacokinetics and below concentrations associated with cytotoxicity, could be a practical adjuvant candidate to improve colistin efficacy and potentially reduce required colistin exposure. Continued evaluation of this combination in appropriately designed pre-clinical and clinical studies is warranted.

### Conclusion

Flufenamic acid, a widely used analgesic, shows practical potential as a colistin adjuvant against colistin-resistant *K. pneumoniae. In vitro*, flufenamic acid restores colistin activity and attenuates resistance emergence, suggesting two potential therapeutic advantages: (i) enabling colistin dose-sparing regimens that may reduce nephrotoxicity and neurotoxicity and (ii) limiting resistance amplification during prolonged exposure. Mechanistic data from membrane permeability assays, transcriptional profiling, and *in silico* modeling are consistent with modulation of the PmrA/PmrB resistance pathway and reduced expression of lipid A-modification genes, providing a plausible mechanistic rationale for the combination while recognizing that direct biochemical target validation was not performed. Given the observed potentiating concentrations *in vitro* and the repurposing feasibility of flufenamic acid, further pre-clinical and clinical studies are warranted to confirm efficacy, safety, and dosing strategies for the flufenamic acid-colistin combination.

## Data Availability

All data generated or analyzed during this study are included in this published article.

## References

[1] Antimicrobial Resistance Collaborators. 2022. Global burden of bacterial antimicrobial resistance in 2019: a systematic analysis. Lancet 399:629–655. doi:10.1016/S0140-6736(21)02724-0 37500506

[2] Naylor NR, Hasso-Agopsowicz M, Kim C, Ma Y, Frost I, Abbas K, Aguilar G, Fuller N, Robotham JV, Jit M. 2025. The global economic burden of antibiotic-resistant infections and the potential impact of bacterial vaccines: a modelling study. BMJ Glob Health 10:e016249. doi:10.1136/bmjgh-2024-016249 PMC12182023 40537271

[3] Macesic N, Uhlemann AC, Peleg AY. 2025. Multidrug-resistant Gram-negative bacterial infections. Lancet 405:257–272. doi:10.1016/S0140-6736(24)02081-6 39826970

[4] Yang S, Wang H, Zhao D, Zhang S, Hu C. 2024. Polymyxins: recent advances and challenges. Front Pharmacol 15:1424765. doi:10.3389/fphar.2024.1424765 38974043 PMC11224486

[5] Li N, Ebrahimi E, Sholeh M, Dousti R, Kouhsari E. 2025. A systematic review and meta-analysis: rising prevalence of colistin resistance in ICU-acquired Gram-negative bacteria. APMIS 133:e13508. doi:10.1111/apm.13508 39710513

[6] Xiao G, Li J, Sun Z. 2023. The combination of antibiotic and non-antibiotic compounds improves antibiotic efficacy against multidrug-resistant bacteria. Int J Mol Sci 24:15493. doi:10.3390/ijms242015493 37895172 PMC10607837

[7] Kumar V, Yasmeen N, Pandey A, Ahmad Chaudhary A, Alawam AS, Ahmad Rudayni H, Islam A, Lakhawat SS, Sharma PK, Shahid M. 2023. Antibiotic adjuvants: synergistic tool to combat multi-drug resistant pathogens. Front Cell Infect Microbiol 13:1293633. doi:10.3389/fcimb.2023.1293633 38179424 PMC10765517

[8] Rahmat Ullah S, Jamal M, Rahman A, Andleeb S. 2024. Comprehensive insights into Klebsiella pneumoniae: unravelling clinical impact, epidemiological trends and antibiotic-resistance challenges. J Antimicrob Chemother 79:1484–1492. doi:10.1093/jac/dkae184 38832539

[9] Poirel L, Jayol A, Nordmann P. 2017. Polymyxins: antibacterial activity, susceptibility testing, and resistance mechanisms encoded by plasmids or chromosomes. Clin Microbiol Rev 30:557–596. doi:10.1128/CMR.00064-16 28275006 PMC5355641

[10] Cannatelli A, Giani T, D’Andrea MM, Di Pilato V, Arena F, Conte V, Tryfinopoulou K, Vatopoulos A, Rossolini GM, COLGRIT Study Group. 2014. MgrB inactivation is a common mechanism of colistin resistance in KPC-producing Klebsiella pneumoniae of clinical origin. Antimicrob Agents Chemother 58:5696–5703. doi:10.1128/AAC.03110-14 25022583 PMC4187966

[11] Luo Q, Wang Y, Xiao Y. 2020. Prevalence and transmission of mobilized colistin resistance (mcr) gene in bacteria common to animals and humans. Biosafety and Health 2:71–78. doi:10.1016/j.bsheal.2020.05.001

[12] Basu S, Veeraraghavan B, Anbarasu A. 2024. Impact of PmrB mutations on clinical Klebsiella pneumoniae with variable colistin-susceptibilities: structural insights and potent therapeutic solutions. Chem Biol Drug Des 103:e14381. doi:10.1111/cbdd.14381 37875387

[13] Elias R, Duarte A, Perdigão J. 2021. A molecular perspective on colistin and Klebsiella pneumoniae: mode of action, resistance genetics, and phenotypic susceptibility. Diagnostics (Basel) 11:1165. doi:10.3390/diagnostics11071165 34202395 PMC8305994

[14] Hondros AD, Young MM, Jaimes FE, Kinkead J, Thompson RJ, Melander C, Cavanagh J. 2025. Two-component system sensor kinase inhibitors target the ATP-lid of PmrB to disrupt colistin resistance in Acinetobacter baumannii. Biochemistry 64:1317–1327. doi:10.1021/acs.biochem.4c00789 40056100 PMC12517351

[15] Vázquez J, López M, Gibert E, Herrero E, Luque FJ. 2020. Merging ligand-based and structure-based methods in drug discovery: an overview of combined virtual screening approaches. Molecules 25:4723. doi:10.3390/molecules25204723 33076254 PMC7587536

[16] Meng XY, Zhang HX, Mezei M, Cui M. 2011. Molecular docking: a powerful approach for structure-based drug discovery. Curr Comput Aided Drug Des 7:146–157. doi:10.2174/157340911795677602 21534921 PMC3151162

[17] Wishart DS, Feunang YD, Guo AC, Lo EJ, Marcu A, Grant JR, Sajed T, Johnson D, Li C, Sayeeda Z, Assempour N, Iynkkaran I, Liu Y, Maciejewski A, Gale N, Wilson A, Chin L, Cummings R, Le D, Pon A, Knox C, Wilson M. 2018. DrugBank 5.0: a major update to the DrugBank database for 2018. Nucleic Acids Res 46:D1074–D1082. doi:10.1093/nar/gkx1037 29126136 PMC5753335

[18] Lipinski CA, Lombardo F, Dominy BW, Feeney PJ. 2001. Experimental and computational approaches to estimate solubility and permeability in drug discovery and development settings. Adv Drug Deliv Rev 46:3–26. doi:10.1016/s0169-409x(00)00129-0 11259830

[19] Wang S. 2012. Bacterial Two-component systems: structures and signaling mechanisms, p 439–466. *In* Huang c (ed), Protein phosphorylation in human health. Intech;2012, Rijeka.

[20] O’Boyle NM, Banck M, James CA, Morley C, Vandermeersch T, Hutchison GR. 2011. Open Babel: an open chemical toolbox. J Cheminform 3:33. doi:10.1186/1758-2946-3-33 21982300 PMC3198950

[21] Willett P. 2006. Similarity-based virtual screening using 2D fingerprints. Drug Discov Today 11:1046–1053. doi:10.1016/j.drudis.2006.10.005 17129822

[22] Webb B, Sali A. 2016. Comparative protein structure modeling using MODELLER. Curr Protoc Bioinformatics 54:5. doi:10.1002/cpbi.3 PMC5031415 27322406

[23] Eberhardt J, Santos-Martins D, Tillack AF, Forli S. 2021. AutoDock Vina 1.2.0: new docking methods, expanded force field, and python bindings. J Chem Inf Model 61:3891–3898. doi:10.1021/acs.jcim.1c00203 34278794 PMC10683950

[24] Murray CW, Erlanson DA, Hopkins AL, Keserü GM, Leeson PD, Rees DC, Reynolds CH, Richmond NJ. 2014. Validity of ligand efficiency metrics. ACS Med Chem Lett 5:616–618. doi:10.1021/ml500146d 24944729 PMC4060940

[25] Tille PM. 2025. Bailey & scott’s diagnostic microbiology. 16th ed. Elsevier, St. Louis (MO).

[26] Cannatelli A, Principato S, Colavecchio OL, Pallecchi L, Rossolini GM. 2018. Synergistic activity of colistin in combination with resveratrol against colistin-resistant Gram-negative pathogens. Front Microbiol 9:1808. doi:10.3389/fmicb.2018.01808 30131787 PMC6091244

[27] Odds FC. 2003. Synergy, antagonism, and what the chequerboard puts between them. J Antimicrob Chemother 52:1. doi:10.1093/jac/dkg301 12805255

[28] Guo T, Li M, Sun X, Wang Y, Yang L, Jiao H, Li G. 2021. Synergistic activity of capsaicin and colistin against colistin-resistant Acinetobacter baumannii: in vitro/vivo efficacy and mode of action. Front Pharmacol 12:744494. doi:10.3389/fphar.2021.744494 34603057 PMC8484878

[29] Pollard JE, Snarr J, Chaudhary V, Jennings JD, Shaw H, Christiansen B, Wright J, Jia W, Bishop RE, Savage PB. 2012. In vitro evaluation of the potential for resistance development to ceragenin CSA-13. J Antimicrob Chemother 67:2665–2672. doi:10.1093/jac/dks276 22899801 PMC3468081

[30] Jangra M, Randhawa HK, Kaur M, Srivastava A, Maurya N, Patil PP, Jaswal P, Arora A, Patil PB, Raje M, Nandanwar H. 2018. Purification, characterization and in vitro evaluation of polymyxin a from Paenibacillus dendritiformis: an underexplored member of the polymyxin family. Front Microbiol 9:2864. doi:10.3389/fmicb.2018.02864 30532748 PMC6265310

[31] Xiao X, Zeng F, Li R, Liu Y, Wang Z. 2022. Subinhibitory concentration of colistin promotes the conjugation frequencies of mcr-1 and bla_NDM-5_-positive plasmids. Microbiol Spectr 10:e0216021. doi:10.1128/spectrum.02160-21 35230128 PMC9045390

[32] Livak KJ, Schmittgen TD. 2001. Analysis of relative gene expression data using real-time quantitative PCR and the 2^−ΔΔC_T_^ method. Methods 25:402–408. doi:10.1006/meth.2001.1262 11846609

[33] Cannatelli A, Di Pilato V, Giani T, Arena F, Ambretti S, Gaibani P, D’Andrea MM, Rossolini GM. 2014. In vivo evolution to colistin resistance by PmrB sensor kinase mutation in KPC-producing Klebsiella pneumoniae is associated with low-dosage colistin treatment. Antimicrob Agents Chemother 58:4399–4403. doi:10.1128/AAC.02555-14 24841267 PMC4136067

[34] Azam M, Gaind R, Yadav G, Sharma A, Upmanyu K, Jain M, Singh R. 2021. Colistin resistance among multiple sequence types of Klebsiella pneumoniae is associated with diverse resistance mechanisms: a report from India. Front Microbiol 12:609840. doi:10.3389/fmicb.2021.609840 33692764 PMC7937630

[35] Gomes AÉI, Stuchi LP, Siqueira NMG, Henrique JB, Vicentini R, Ribeiro ML, Darrieux M, Ferraz LFC. 2018. Selection and validation of reference genes for gene expression studies in Klebsiella pneumoniae using reverse transcription quantitative real-time PCR. Sci Rep 8:9001. doi:10.1038/s41598-018-27420-2 29899556 PMC5998039

[36] Anuwongcharoen N, Phanus-Umporn C, Chatupheeraphat C, Weakwiweak K, Kaewsai N, Eiamphungporn W. 2025. Insight into the PmrB structures of colistin-resistant Gram-negative bacteria through the multi-template ligand-guided homology modeling and in silico mutagenesis. PeerJ 13:e19945. doi:10.7717/peerj.19945 40936770 PMC12422264

[37] Ravindranath PA, Forli S, Goodsell DS, Olson AJ, Sanner MF. 2015. AutoDockFR: advances in protein-ligand docking with explicitly specified binding site flexibility. PLoS Comput Biol 11:e1004586. doi:10.1371/journal.pcbi.1004586 26629955 PMC4667975

[38] Abraham M, Alekseenko A, Andrews B, Basov V, Bauer P, Bird H, et al.. 2025. GROMACS. Zenodo, Manual Geneva.

[39] Valdés-Tresanco MS, Valdés-Tresanco ME, Valiente PA, Moreno E. 2021. gmx_MMPBSA: a new tool to perform end-state free energy calculations with GROMACS. J Chem Theory Comput 17:6281–6291. doi:10.1021/acs.jctc.1c00645 34586825

[40] Delgado J, Radusky LG, Cianferoni D, Serrano L. 2019. FoldX 5.0: working with RNA, small molecules and a new graphical interface. Bioinformatics 35:4168–4169. doi:10.1093/bioinformatics/btz184 30874800 PMC6792092

[41] Chatupheeraphat C, Kaewsai N, Anuwongcharoen N, Phanus-umporn C, Pornsuwan S, Eiamphungporn W. 2025. Penfluridol synergizes with colistin to reverse colistin resistance in Gram-negative bacilli. Sci Rep 15:16114. doi:10.1038/s41598-025-01303-9 40341530 PMC12062240

[42] Hua Y, Dai X, Xu Y, Xing G, Liu H, Lu T, Chen Y, Zhang Y. 2022. Drug repositioning: progress and challenges in drug discovery for various diseases. Eur J Med Chem 234:114239. doi:10.1016/j.ejmech.2022.114239 35290843 PMC8883737

[43] Ghirga F, Stefanelli R, Cavinato L, Lo Sciuto A, Corradi S, Quaglio D, Calcaterra A, Casciaro B, Loffredo MR, Cappiello F, Morelli P, Antonelli A, Rossolini GM, Mangoni M, Mancone C, Botta B, Mori M, Ascenzioni F, Imperi F. 2020. A novel colistin adjuvant identified by virtual screening for ArnT inhibitors. J Antimicrob Chemother 75:2564–2572. doi:10.1093/jac/dkaa200 32514531 PMC7443731

[44] Tang F, Peng W, Kou X, Chen Z, Zhang L. 2024. High-throughput screening identification of apigenin that reverses the colistin resistance of mcr-1-positive pathogens. Microbiol Spectr 12:e0034124. doi:10.1128/spectrum.00341-24 39248524 PMC11448233

[45] McPhee JB, Lewenza S, Hancock REW. 2003. Cationic antimicrobial peptides activate a two-component regulatory system, PmrA-PmrB, that regulates resistance to polymyxin B and cationic antimicrobial peptides in Pseudomonas aeruginosa. Mol Microbiol 50:205–217. doi:10.1046/j.1365-2958.2003.03673.x 14507375

[46] Pushpakom S, Iorio F, Eyers PA, Escott KJ, Hopper S, Wells A, Doig A, Guilliams T, Latimer J, McNamee C, Norris A, Sanseau P, Cavalla D, Pirmohamed M. 2019. Drug repurposing: progress, challenges and recommendations. Nat Rev Drug Discov 18:41–58. doi:10.1038/nrd.2018.168 30310233

[47] Ashburn TT, Thor KB. 2004. Drug repositioning: identifying and developing new uses for existing drugs. Nat Rev Drug Discov 3:673–683. doi:10.1038/nrd1468 15286734

[48] López-Pérez K, Avellaneda-Tamayo JF, Chen L, López-López E, Juárez-Mercado KE, Medina-Franco JL, Miranda-Quintana RA. 2024. Molecular similarity: theory, applications, and perspectives. Artif Intell Chem 2:100077. doi:10.1016/j.aichem.2024.100077 40124654 PMC11928018

[49] Torres J, Lo Y-C. 2016. Chemical similarity networks for drug discovery, p 53–72. *In* Chen T, Chai SC (ed), Special topics in drug discovery. IntechOpen, London.

[50] Bajusz D, Rácz A, Héberger K. 2015. Why is Tanimoto index an appropriate choice for fingerprint-based similarity calculations? J Cheminform 7:20. doi:10.1186/s13321-015-0069-3 26052348 PMC4456712

[51] Baldi P, Nasr R. 2010. When is chemical similarity significant? The statistical distribution of chemical similarity scores and its extreme values. J Chem Inf Model 50:1205–1222. doi:10.1021/ci100010v 20540577 PMC2914517

[52] Ferreira LG, Dos Santos RN, Oliva G, Andricopulo AD. 2015. Molecular docking and structure-based drug design strategies. Molecules 20:13384–13421. doi:10.3390/molecules200713384 26205061 PMC6332083

[53] Dotolo S, Cervellera C, Russo M, Russo GL, Facchiano A. 2021. Virtual screening of natural compounds as potential PI_3_K-AKT1 signaling pathway inhibitors and experimental validation. Molecules 26:492. doi:10.3390/molecules26020492 33477701 PMC7831918

[54] Gryniukova A, Kaiser F, Myziuk I, Alieksieieva D, Leberecht C, Heym PP, Tarkhanova OO, Moroz YS, Borysko P, Haupt VJ. 2023. AI-powered virtual screening of large compound libraries leads to the discovery of novel inhibitors of sirtuin-1. J Med Chem 66:10241–10251. doi:10.1021/acs.jmedchem.3c00128 37499195

[55] Jang WD, Jeon S, Kim S, Lee SY. 2021. Drugs repurposed for COVID-19 by virtual screening of 6,218 drugs and cell-based assay. Proc Natl Acad Sci USA 118:e2024302118. doi:10.1073/pnas.2024302118 34234012 PMC8325362

[56] Soteras Gutiérrez I, Lin F-Y, Vanommeslaeghe K, Lemkul JA, Armacost KA, Brooks CL III, MacKerell AD. 2016. Parametrization of halogen bonds in the CHARMM general force field: improved treatment of ligand-protein interactions. Bioorg Med Chem 24:4812–4825. doi:10.1016/j.bmc.2016.06.034 27353885 PMC5053860

[57] Sundy JS. 2001. COX-2 inhibitors in rheumatoid arthritis. Curr Rheumatol Rep 3:86–91. doi:10.1007/s11926-001-0055-9 11177775

[58] Chavez-Dozal AA, Jahng M, Rane HS, Asare K, Kulkarny VV, Bernardo SM, Lee SA. 2014. In vitro analysis of flufenamic acid activity against Candida albicans biofilms. Int J Antimicrob Agents 43:86–91. doi:10.1016/j.ijantimicag.2013.08.018 24156913 PMC3902125

[59] Seong YJ, Alhashimi M, Mayhoub A, Mohammad H, Seleem MN. 2020. Repurposing fenamic acid drugs to combat multidrug-resistant Neisseria gonorrhoeae. Antimicrob Agents Chemother 64:e02206-19. doi:10.1128/AAC.02206-19 32393483 PMC7318036

[60] Zhang S, Tang H, Wang Y, Nie B, Yang H, Yuan W, Qu X, Yue B. 2020. Antibacterial and antibiofilm effects of flufenamic acid against methicillin-resistant Staphylococcus aureus. Pharmacol Res 160:105067. doi:10.1016/j.phrs.2020.105067 32650057

[61] Preethi B, Shanthi V, Ramanathan K. 2016. Identification of potential therapeutics to conquer drug resistance in Salmonella Typhimurium: drug repurposing strategy. BioDrugs 30:593–605. doi:10.1007/s40259-016-0200-7 27761807

[62] Zhang Y, Han Y, Wang L, Kong J, Pan W, Zhang X, Chen L, Yao Z, Zhou T, Cao J. 2023. Flufenamic acid, a promising agent for the sensitization of colistin-resistant gram-negative bacteria to colistin. Microbiol Spectr 11:e0405222. doi:10.1128/spectrum.04052-22 36971552 PMC10100705

[63] Clinical and Laboratory Standards Institute (CLSI). 2025. Performance standards for antimicrobial susceptibility testing. *In* CLSI supplement M100, 35th ed. Clinical and Laboratory Standards Institute, Wayne (PA).

[64] The European Committee on Antimicrobial Susceptibility Testing (EUCAST). 2025. Breakpoint tables for interpretation of MICs and zone diameters. Version 15.0. Växjö, Sweden: EUCAST. Available from: https://www.eucast.org/fileadmin/eucast/pdf/breakpoints/v_15.0_Breakpoint_Tables.pdf

[65] Gogry FA, Siddiqui MT, Sultan I, Haq QMR. 2021. Current update on intrinsic and acquired colistin resistance mechanisms in bacteria. Front Med (Lausanne) 8:677720. doi:10.3389/fmed.2021.677720 34476235 PMC8406936

[66] Kathayat D, Antony L, Deblais L, Helmy YA, Scaria J, Rajashekara G. 2020. Small molecule adjuvants potentiate colistin activity and attenuate resistance development in Escherichia coli by affecting pmrAB System. Infect Drug Resist 13:2205–2222. doi:10.2147/IDR.S260766 32764996 PMC7360418

[67] Harris TL, Worthington RJ, Hittle LE, Zurawski DV, Ernst RK, Melander C. 2014. Small molecule downregulation of PmrAB reverses lipid A modification and breaks colistin resistance. ACS Chem Biol 9:122–127. doi:10.1021/cb400490k 24131198

[68] Yoon EJ, Mo JW, Kim JW, Jeong MC, Yoo JS. 2024. Alteration in the morphological and transcriptomic profiles of Acinetobacter baumannii after exposure to colistin. Microorganisms 12:1644. doi:10.3390/microorganisms12081644 39203486 PMC11356899

[69] Olaitan AO, Morand S, Rolain JM. 2014. Mechanisms of polymyxin resistance: acquired and intrinsic resistance in bacteria. Front Microbiol 5:643. doi:10.3389/fmicb.2014.00643 25505462 PMC4244539

[70] Cheng HY, Chen YF, Peng HL. 2010. Molecular characterization of the PhoPQ-PmrD-PmrAB mediated pathway regulating polymyxin B resistance in Klebsiella pneumoniae CG43. J Biomed Sci 17:60. doi:10.1186/1423-0127-17-60 20653976 PMC2919465

[71] Xu C, Zhang Y, Ma L, Zhang G, Li C, Zhang C, Li Y, Zeng X, Li Y, Dong N. 2024. Valnemulin restores colistin sensitivity against multidrug-resistant gram-negative pathogens. Commun Biol 7:1122. doi:10.1038/s42003-024-06805-2 39261709 PMC11390741

[72] Zhong ZX, Zhou S, Liang YJ, Wei YY, Li Y, Long TF, He Q, Li MY, Zhou YF, Yu Y, Fang LX, Liao XP, Kreiswirth BN, Chen L, Ren H, Liu YH, Sun J. 2023. Natural flavonoids disrupt bacterial iron homeostasis to potentiate colistin efficacy. Sci Adv 9:eadg4205. doi:10.1126/sciadv.adg4205 37294761 PMC10256158

[73] Lentjes EG, van Ginneken CA. 1987. Pharmacokinetics of flufenamic acid in man. Int J Clin Pharmacol Ther Toxicol 25:185–187. 3583467

[74] Wagner H, Kostka KH, Lehr CM, Schaefer UF. 2002. Human skin penetration of flufenamic acid: in vivo/in vitro correlation (deeper skin layers) for skin samples from the same subject. J Invest Dermatol 118:540–544. doi:10.1046/j.0022-202x.2001.01688.x 11874496

[75] Kamour A, Crichton S, Cooper G, Lupton DJ, Eddleston M, Vale JA, Thompson JP, Thomas SHL. 2017. Central nervous system toxicity of mefenamic acid overdose compared with other NSAIDs: an analysis of cases reported to the United Kingdom National Poisons Information Service. Brit J Clinical Pharma 83:855–862. doi:10.1111/bcp.13169 PMC5346865 27785820

